# Oleanolic Acid Induces Differentiation of Neural Stem Cells to Neurons: An Involvement of Transcription Factor Nkx-2.5

**DOI:** 10.1155/2015/672312

**Published:** 2015-07-09

**Authors:** You Ning, Jianhua Huang, Bill Kalionis, Qin Bian, Jingcheng Dong, Junzhen Wu, Xiantao Tai, Shijin Xia, Ziyin Shen

**Affiliations:** ^1^Institute of Integrated Traditional Chinese Medicine and Western Medicine, Huashan Hospital, Fudan University, Shanghai 200040, China; ^2^Department of Obstetrics and Gynaecology and Department of Perinatal Medicine Pregnancy Research Centre, Royal Women's Hospital, University of Melbourne, Parkville, VIC 3052, Australia; ^3^Shanghai Institute of Geriatrics, Huadong Hospital, Fudan University, Shanghai 200040, China; ^4^School of Acupuncture, Massage and Rehabilitation, Yunnan University of Traditional Chinese Medicine, Kunming 650500, China

## Abstract

Neural stem cells (NSCs) harbor the potential to differentiate into neurons, astrocytes, and oligodendrocytes under normal conditions and/or in response to tissue damage. NSCs open a new way of treatment of the injured central nervous system and neurodegenerative disorders. Thus far, few drugs have been developed for controlling NSC functions. Here, the effect as well as mechanism of oleanolic acid (OA), a pentacyclic triterpenoid, on NSC function was investigated. We found OA significantly inhibited neurosphere formation in a dose-dependent manner and achieved a maximum effect at 10 nM. OA also reduced 5-ethynyl-2′-deoxyuridine (EdU) incorporation into NSCs, which was indicative of inhibited NSC proliferation. Western blotting analysis revealed the protein levels of neuron-specific marker tubulin-*β*III (TuJ1) and Mash1 were increased whilst the astrocyte-specific marker glial fibrillary acidic protein (GFAP) decreased. Immunofluorescence analysis showed OA significantly elevated the percentage of TuJ1-positive cells and reduced GFAP-positive cells. Using DNA microarray analysis, 183 genes were differentially regulated by OA. Through transcription factor binding site analyses of the upstream regulatory sequences of these genes, 87 genes were predicted to share a common motif for Nkx-2.5 binding. Finally, small interfering RNA (siRNA) methodology was used to silence Nkx-2.5 expression and found silence of Nkx-2.5 alone did not change the expression of TuJ-1 and the percentage of TuJ-1-positive cells. But in combination of OA treatment and silence of Nkx-2.5, most effects of OA on NSCs were abolished. These results indicated that OA is an effective inducer for NSCs differentiation into neurons at least partially by Nkx-2.5-dependent mechanism.

## 1. Introduction

Oleanolic acid (OA) is a pentacyclic triterpenoid extensively found in a variety of plants and medicinal herbs such as* Olea europaea*,* Viscum album *L., and* Ligustrum lucidum* [1]. The original plant of* Ligustrum lucidum* has been used to treat multiple diseases with particular symptom profiles in traditional Chinese medicine for more than thousand years [2]. As a main effective component of* Ligustrum lucidum*, OA is attributed to possess a wide range of activities including anticancer [3–5], anti-inflammatory [6], hepatoprotective [7, 8], nephroprotective [9], and antidiabetic [10, 11] properties, thereby displaying promising clinical application. Multiple molecular targets or signaling pathways are involved in the mechanism of OA action. OA was reported to inhibit nuclear factor kappa B (NF*κ*B) activation and nuclear translocation, resulting in suppression of the tumor necrosis factor-alpha-induced inflammatory response [12]. OA is an inhibitor of glycogen phosphorylase [13, 14], which catalyzes the key step in the generation of glucose from glycogen. Researchers also showed that OA may act through farnesoid x receptor (FXR) to selectively regulate FXR target genes and thus mediate some of its beneficial effects [15]. Nuclear factor erythroid 2-related factor (Nrf2), a transcription factor that induces various antioxidant and cytoprotective genes, is implicated in the hepatoprotective effects of OA [7, 16]. Recently, derivatives and homologues of OA were reported to significantly improve spatial memory retention and reduced plaque burden in an Alzheimer's mice model [17]. Since NSCs play an important role in pathogenesis of Alzheimer and hold great promises for its treatment, in the present study, we investigated the effects of OA on NSCs.

Neural stem cells (NSCs) are a type of stem cell residing in the central nervous system and spinal cord. They have the potential to differentiate into neurons, astrocytes, and oligodendrocytes under normal conditions and/or in response to tissue damage. NSCs represent a novel means of treating the injured central nervous system as well as neurodegenerative disorders [18]. The factors maintaining the self-renewal capacity of NSCs have been widely studied. The Notch signaling pathway plays a pivotal role in maintaining the NSC pool [19]. Bone morphogenetic proteins (BMPs) [20] and inhibitory basic helix loop helix (bHLH) transcription factors [21] also contribute to the regulation of self-renewal of NSCs. The neurotrophin family of factors are important inducers of NSC differentiation [22]. Other factors including sonic hedgehog (Shh) [23], retinoic acid (RA) [24], and neuropathiazol [25] also significantly increase neuronal differentiation of NSCs* in vitro*. Factors that drive the differentiation of NSCs into astrocytes include leukemia inhibitory factor (LIF) and neuropoietin (NP), ciliary neurotrophic factor (CNTF), endothelial growth factor (EGF), and members of the BMP family [26–29]. Although great advances have been made in revealing the molecular basis for NSC function, few drugs have been developed that control NSC fate.

In the present study, we tested the effects of OA on NSC self-renewal and on the multidifferentiative potential of NSCs. To the best of our knowledge, we are the first to show that OA induces the differentiation of NSCs to neurons by Nkx-2.5-dependent mechanism.

## 2. Materials and Methods

### 2.1. Animals

Pregnant Kunming female mice were maintained in animal facility of Public Health Center of Fudan University. All performances on mice were approved by the Animal Care and Use Committee of Fudan University, permit number SCXK(Hu) 2010-0016, and in accordance with the guidelines for animal use of the National Institutes of Health.

### 2.2. NSCs Preparation and Culture

Neurosphere culture was performed as described previously [30] with some modifications. Murine embryos at embryonic day 14 (E14) were collected from timed-pregnant Kunming mice and placed in D-PBS (Invitrogen, CA, USA). The forebrain neuroepithelium was removed from the rest of the embryo under a dissection microscope. The resultant tissue was mechanically dissociated into a single cell suspension with a small-bore, fire-polished Pasteur pipette. The cells were filtered through a sterile nylon mesh and washed twice with DMEM/F12 medium (Invitrogen, CA, USA) containing 100 units/mL penicillin and 100 *μ*g/mL streptomycin. The number of viable cells was determined by staining with Trypan Blue. Neurosphere culture was initiated by seeding cells at a density of 1 × 10^5^ to 2 × 10^5^ viable cells/mL in the basal medium supplemented with 20 ng/mL human recombinant fibroblast growth factor-2 (hrFGF2, Invitrogen, CA, USA), 20 ng/mL human recombinant endothelial growth factor (hrEGF, Invitrogen, CA, USA), and Stempro NSC supplement (Invitrogen, CA, USA).

### 2.3. Neurosphere Formation Assay

Cells were plated under clonal conditions at 5 cells/*μ*L in 96-well (0.1 mL/well) in serum-free DMEM/F12 medium containing 20 ng/mL hrFGF-2 (Invitrogen, CA, USA), Stempro NSC supplement (Invitrogen, CA, USA), and 100 units/mL penicillin and 100 *μ*g/mL streptomycin. The next day, various concentrations of OA (Yousi Biotechnology, Shanghai, China; purity above 99% HPLC) were added into each 96-well plate. The total number of spheres that formed in each well was counted after 8 d. Only colonies >40 *μ*m in diameter were counted as neurospheres. Neurosphere size was determined by measuring the diameters of individual neurospheres under light microscopy and expressed as a volume (assuming a spherical shape). The consecutive second, third, or fourth passages were used to verify neurosphere formation.

### 2.4. Cell Proliferation Assay

Cell proliferation was assayed based on the incorporation of EdU and its subsequent detection by a fluorescent azide through a Cu(I)-catalyzed [3 + 2] cycloaddition reaction (“click” chemistry) as described previously [31]. In brief, single NSCs were grown in the well of 96-well plate in DMEM/F12 medium containing 20 ng/mL hrFGF-2 and hrEGF (Invitrogen, CA, USA), Stempro NSC supplement (Invitrogen, CA, USA), and 100 units/mL penicillin and 100 *μ*g/mL streptomycin. EdU was added to the culture media in a final concentration of 10 *μ*M for 3 h. Cells were fixed by formaldehyde fixation and permeabilized with 0.5% Triton X-100. Cells were then stained by incubating for 30 min with 100 mM Tris, 0.5 mM CuSO_4_, 10 *μ*M Alexa 594-azide, and 50 mM ascorbic acid. Cells were counterstained with 4′-6-diamidino-2-phenylindole (DAPI). The cells were washed and imaged by fluorescence microscopy.

### 2.5. Differentiation Assay

Single NSCs were plated at a density of 5000 cells/well onto 10 *μ*g/mL PDL-coated 96-well culture dishes (Corning, NY, USA) and incubated for 3 d in differentiation medium comprising DMEM/F12 containing 1% fetal bovine serum (Invitrogen, CA, USA), Stempro NSC supplement (Invitrogen, CA, USA), and 100 units/mL penicillin and 100 *μ*g/mL streptomycin. Three days later, cells were harvested for Western blot and immunocytochemistry analysis.

### 2.6. Western Blot Analysis

Cells cultured in differentiation medium were harvested and lysed in a buffer containing 50 mM HEPES-NaOH (pH 7.5), 100 mM KCl, 1% Triton X-100, 1% sodium deoxycholate, 0.1% sodium dodecyl sulfate, 1 mM EGTA, 1 mM dithiothreitol, 1 mM phenylmethylsulfonyl fluoride, 0.5% protease inhibitor cocktail (Sigma-Aldrich, MO, USA), 1 mM Na_3_VO_4_, 10 mM NaF, and 20 mM *β*-glycerophosphate. The resultant extracts were centrifuged at 14,000 g for 15 min at 4°C to obtain clear cell lysates. Protein concentrations were determined using the Biyotime protein assay kit (Beyotime, Shanghai, China) with BSA as a standard. The equivalent of 35 *μ*g protein was loaded in each track and proteins were separated by sodium dodecyl sulfate polyacrylamide gel electrophoresis and transferred to nitrocellulose membranes (Amersham Biosciences, USA). The membranes were blocked with 5% (wt/vol) skim milk in phosphate buffered saline containing 0.1% Tween 20 and blotted with antibodies for tubulin-*β*III (TuJ1) (1 : 200; Chemicon, USA), Mash1 (1 : 200; Chemicon, USA), and glial fibrillary acidic protein (GFAP) (1 : 500; Chemicon, USA), followed by incubation with the appropriate secondary HRP-conjugated goat anti-mouse or -rabbit antibodies (1 : 5000; Jackson ImmunoResearch, USA). Immunoreactive bands were visualized with ECL reagents (Biyotime, Shanghai, China).

### 2.7. Immunocytochemistry

Cells cultured in differentiation medium were fixed for 20 min using 4% paraformaldehyde, blocked in 1% BSA and 0.1% Triton X-100, washed by PBS, incubated for 30 min with 0.3% H_2_O_2_ to inhibit endogenous peroxidases, and then blocked for 1 h using 3% BSA in PBS/0.1% Triton X-100. The following primary antibodies were used and incubated for 2 hours in room temperature: monoclonal mouse anti-TuJ1 (diluted 1 : 200; Chemicon, USA), rabbit anti-GFAP (1 : 500; Chemicon, USA), and rabbit anti-nestin (1 : 1000; Chemicon, USA). Secondary Alexa conjugated 594 F(ab)′2 goat anti-rabbit antibody and 488 Alexa conjugated goat anti-mouse IgG (H + L) (1 : 1000; Invitrogen, USA) were added for 1.5 h in PBS in 1% BSA and 0.1% Triton X-100. Then cells were counterstained with DAPI. The number of immunoreactive cells in each well was counted using a fluorescence microscope.

### 2.8. Microarray and Data Analysis

Cells cultured in differentiation medium for 3 days were harvested and lysed in TRIzol reagent (Invitrogen, USA). Total RNA was isolated using the Qiagen RNeasy kit (Qiagen), according to the manufacturer's protocol. The isolated RNA was subjected to a quality control test. RNA from each sample was used for cDNA synthesis followed by labeling of the cDNA with Cy3. The labeled cDNA samples were submitted to NimbleGen and hybridized to mouse gene expression 12 × 135 K arrays (Roche NimbleGen, 05543797001) that represents 44,170 mouse genes. The single color NimbleGen arrays were scanned with a GenePix 4000B microarray scanner. The data were extracted from scanned images using NimbleScan v2.5 software. Expression data were normalized through quantile normalization and the Robust Multichip Average (RMA) algorithm was included in the NimbleScan software. The Probe level (∗_norm_RMA.pair) files and gene level (∗_RMA.calls) files were generated after normalization. All gene level files were imported into Agilent GeneSpring GX software (version 11.5.1) for further analysis. Differentially expressed genes, hierarchical clustering, pathway analysis, and gene ontology (GO) analysis were performed. For analysis of transcription factor binding sites of differentially expressed genes, the online tool oPOSSUM (http://www.cisreg.ca/oPOSSUM/) was used [32]. All data is MIAME compliant and the raw data as well as processed data have been deposited in GEO database, accession number GSE38394.

### 2.9. Quantitative Real-Time PCR

Total RNA from cells was extracted using TRIzol reagent (Invitrogen, USA). One microgram of total RNA was reverse transcribed using the Advantage RT-for-PCR kit (Qiagen, Valencia, CA). Freshly transcribed cDNA was used for quantitative real-time PCR using SYBR Green (Bio-Rad, Hercules, CA). The primers for each gene were designed by online tool Primer3 (http://frodo.wi.mit.edu/) listed in supplementary Table 1 in Supplementary Material available online at http://dx.doi.org/10.1155/2015/672312. The PCR was carried out in a RotorGene real-time DNA amplification system (Corbett Research, Sydney, Australia) as described in our previous study [33].

### 2.10. RNA Interference

Nkx-2.5 siRNA duplexes for mouse cell application were obtained from Santa Cruz Biotechnology (Catalog. sc-36076). The detailed protocol of siRNA followed the manufacturer's guidelines. Briefly, 2 × 10^4^ cells per well were seeded in 200 *μ*L antibiotic-free differentiation medium of DMEM/F12 containing 1% fetal bovine serum (Invitrogen, CA, USA), Stempro NSC supplement (Invitrogen, CA, USA) in a 96-well tissue culture plate. Two days later, 6 *μ*L of siRNA duplex was diluted into 100 *μ*L siRNA Transfection Medium (Catalog. sc-36868, Santa Cruz Biotechnology); then 5 *μ*L of siRNA Transfection Reagent was diluted (Catalog. sc-29528 Santa Cruz Biotechnology) into 100 *μ*L of siRNA-containing Transfection Medium mixture, which was then added to the cells. The cells were incubated for 6 h at 37°C in a CO_2_ incubator. Then the medium was removed and replaced with antibiotic-free differentiation medium (see Section 2.5). Following a further 48 h incubation period, the cells were harvested and used to perform immunocytochemistry and Western blot analysis. A nonspecific siRNA (Catalog. sc-37007, Santa Cruz Biotechnology) was transfected as a negative control.

### 2.11. Statistical Analysis

Results are expressed as mean ± SD and statistical significance was calculated using a Student *t*-test or analysis of variance by R software. The significance level was defined as *P* < 0.05. The number of replicated experiments is indicated in Results or in the figure legends.

## 3. Results

### 3.1. OA Inhibited the Formation of Neurospheres

**Figure 1:**
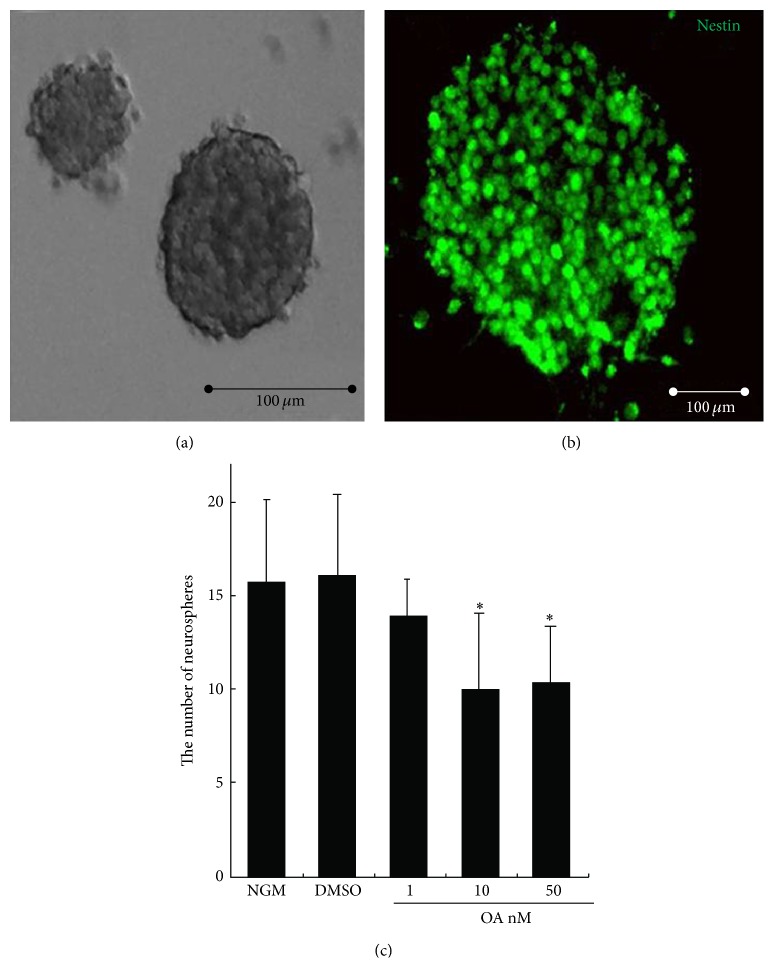
Effects of OA on neurosphere formation of neural stem cells* in vitro*. Single NSCs at a density of 500 cells/well were cultured in normal growth medium (NGM) containing DMEM/F12 supplemented with hrFGF for 7 days to form various sizes of neurospheres (a). The neurospheres expressed the NSC marker nestin (b). Single NSCs were exposed to NGM, DMSO (0.1%), and 1, 10, and 50 nM OA dissolved in DMSO (0.1%), respectively. OA caused a significant decrease in frequency of neurosphere formation (c). Scale bars: 100 *μ*m. Results were expressed as mean ± SD of six independent experiments. ^*^ *P* < 0.05 versus NGM.

Neurosphere formation reflects the self-renewal capacity of NSCs when single NSCs are plated at a very low cell density. In our growth culturing conditions, NSCs formed neurospheres of various sizes with diameters ranging from 20 *μ*m to more than 100 *μ*m (Figure 1(a)). These neurospheres stained positive for nestin, a well-known NSC marker (Figure 1(b)), providing evidence of NSC and/or neural progenitor identity. Next, the frequency of neurosphere formation was calculated with or without added OA. The control group formed 15.75 ± 4.43 (*n* = 6) neurospheres from 500 initially seeded cells, resulting in a frequency of about 3.15%. The vehicle solvent DMSO did not significantly change the frequency of neurosphere formation (16.5 ± 3.99, *n* = 6). After addition of OA, the neurosphere frequency for low (1 nM), middle (10 nM), and high (50 nM) concentrations of OA was 14 ± 1.91, 10 ± 4.08, and 10.43 ± 2.94 (*n* = 6), respectively. Compared with the control, 10 and 50 nM OA significantly decreased neurosphere formation of NSCs (*P* < 0.05). The maximum inhibitory effect of OA was achieved at a concentration of 10 nM (Figure 1(c)).

### 3.2. OA Inhibited the Proliferation of NSCs

**Figure 2:**
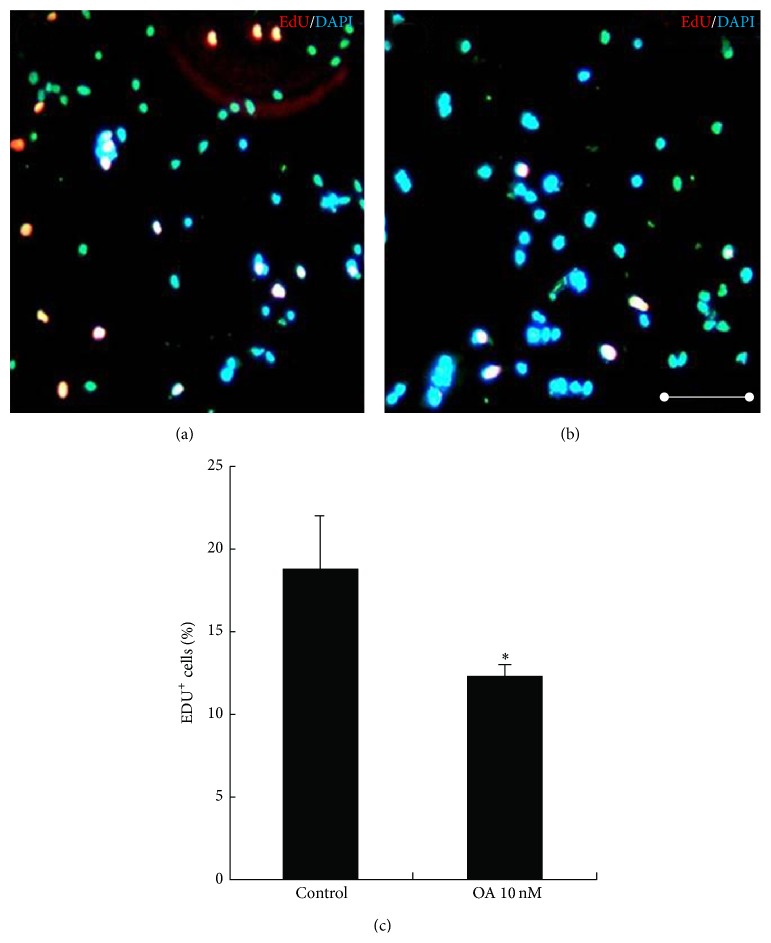
Effects of OA on the proliferation of NSCs. Single NSCs were plated at a density of 5000 cells per well in PDL-coated 96-well plate for 12 h. Then, cells were subjected to 10 nM EdU for 2 h, followed by addition of 10 nM OA (b) or without added OA (a). Then EdU immunofluorescence analysis was performed. The cell nuclei were counterstained with DAPI. The percentage of EdU-positive cells in a total of 1000 cells was calculated. OA significantly inhibited DNA incorporation (c). Scale bars: 100 *μ*m. Results were expressed as mean ± SD from four independent experiments. ^*^ *P* < 0.05 versus control.

Decreased neurosphere formation (Figure 1(c)) may be due to compromised NSC cell proliferation. NSC proliferation was investigated following addition of OA using EdU incorporation, allowing the index of cells in S phase of cell cycle to be determined. In the control, the ratio of EdU-positive cells to total cells was 18.8 ± 3.2% (*n* = 4) (Figure 2(a)). In the presence of OA at 10 nM, the incorporation of EdU into NSCs decreased significantly (12.4 ± 0.6%, *n* = 4; *P* < 0.05 versus control) (Figures 2(b) and 2(c)).

### 3.3. OA Induced the Differentiation of NSCs to Neurons

**Figure 3:**
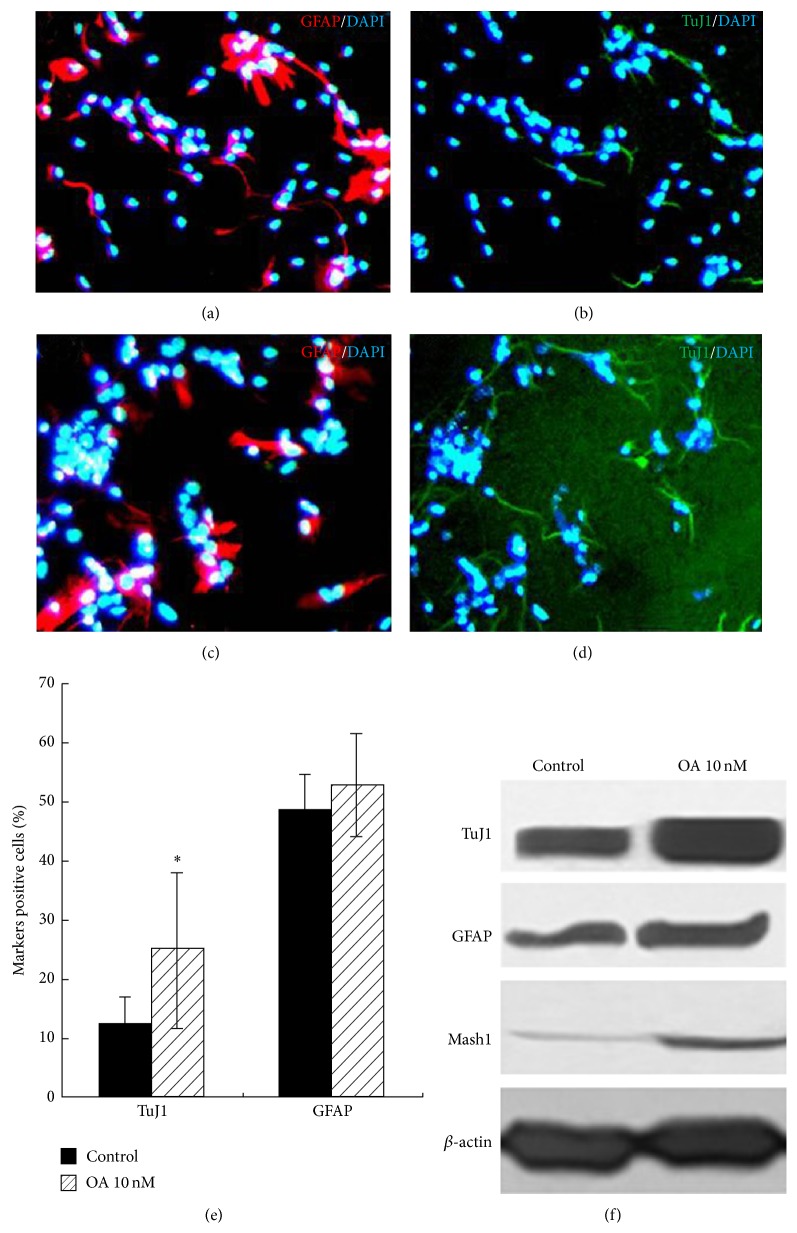
Effects of OA on the differentiation of NSCs. Single NSCs were seeded at a density of 50 cells/*μ*L in PDL-coated 96-well plate in differentiation medium for 48 h without OA (a, b) or with 10 nM OA (c, d). Cells were incubated with primary antibodies to TuJ1 and GFAP and the corresponding secondary antibodies and visualized with Alexa-conjugated 594 F(ab)′2 goat anti-rabbit antibody and 488 Alexa-conjugated goat anti mouse IgG (H + L). The ratio of TuJ1, GFAP-positive cells against DAPI-stained cells was calculated. OA significantly increased the TuJ1-positive cells and decreased the GFAP-positive cells (e). The same cells were performed using Western blotting. OA significantly increased TuJ1, Mash1, and GFAP protein expression (f). Scale bars: 100 *μ*m. Results were expressed as mean ± SD from three independent experiments. ^*^ *P* < 0.05 versus control.

When single cells were cultured in a monolayer on the surface of PDL-coated dishes in serum without growth factors for 48 h, immunochemistry results showed that, in control group, 47.65 ± 4.3% (*n* = 4) cells were GFAP-positive (Figure 3(a)) and 12.45 ± 4.5% (*n* = 4) cells were TuJ1-positive (Figure 3(b)), indicating that NSCs were multipotent. After treatment with OA, the percentage of GFAP-positive cells did not change (*P* > 0.05 versus control) (Figure 3(c)), but the percentage of TuJ1-positive cells significantly increased to 24.93 ± 6.19% (*n* = 4; *P* < 0.05 versus control) (Figures 3(d) and 3(e)). Western blotting analysis of total cell lysates of the OA-treated or untreated cells showed an increased expression of TuJ1, a neuron-specific marker and Mash1, and a neuron-specific transcription factor following OA treatment (Figure 3(f)). The protein expression of GFAP, an astrocyte-specific marker, showed a slight increase (Figure 3(f)). Results indicated OA as a differentiation inducer for NSCs to neuron.

### 3.4. Identification of Differentially Expressed Genes Induced by OA

**Figure 4:**
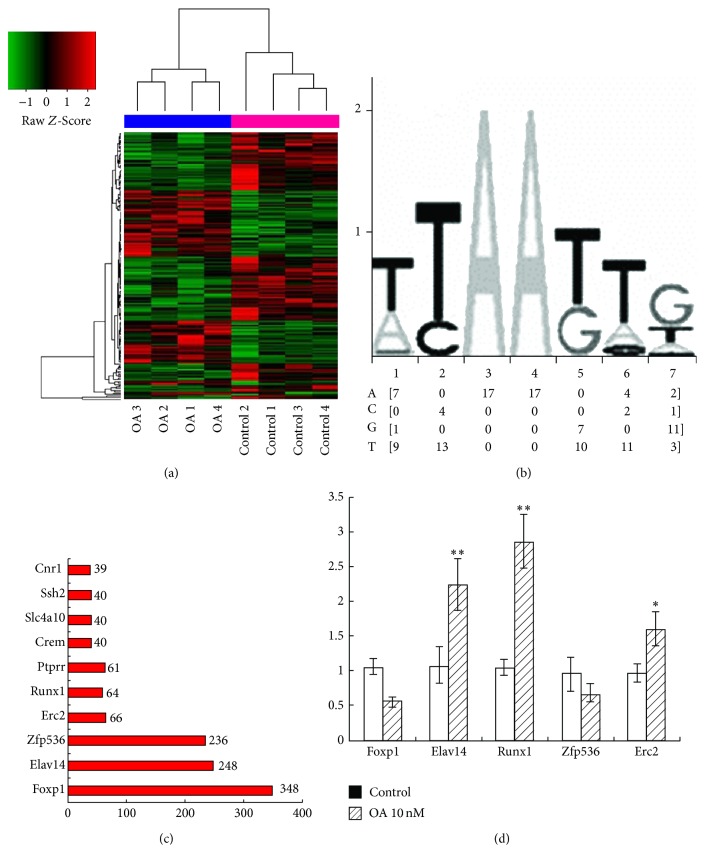
Genes differentially regulated by OA and bioinformatical analysis of these genes. Single NSCs at density of 5000 cells/well in 6-well plate were cultured in differentiation medium. Cells were exposed to 10 nM OA or not. 48 h later, cells were harvested. The total mRNA was extracted followed by whole genome mRNA expression measurement. 183 genes were differentially regulated by OA. Among 183 genes, 87 were predicted under the control of Nkx-2.5. Using these 87 genes, hierarchical clustering analysis was performed (a). The upstream regulation sequences among these 87 genes shared common predicted motif for Nkx-2.5 binding. The frequency matrix for this motif was shown. TTAATTG was the most frequent pattern (b). The number of binding sites existing in the upstream regulatory sequence of 87 genes was calculated. Top 10 genes with the largest numbers were shown (c). The expression of 5 differentially regulated genes including Foxp1, Elavl4, Runx1, Znf536, and Erc2 was confirmed by quantitative real-time PCR (d).

NSCs were cultured in differentiation medium and four independent biological replicates of gene expression profiling experiments for each group were conducted. After treatment with OA, there were 80 genes upregulated and 103 genes downregulated by more than 1.5-fold and with a *P* value < 0.05. These genes were enriched in several pathways including the T cell receptor signaling pathway, intestinal immune network for IgA production, allograft rejection, and autoimmune thyroid disease (supplementary Table 2). Gene ontology (GO) enrichment for differentially regulated genes also identified a number of potential pathways (supplementary Table 3). Because of the important role of transcription factors in stem cell self-renewal and differentiation, we predicted the possible upstream sites for transcription factor binding in 183 differentially expressed genes. We noted that the transcription factor Nkx-2.5 was predicted to control up to 87 target genes (supplementary Table 4). With the expression of these 87 genes, a clustering analysis successfully classified samples into two groups, which correlated well with changes in the control group and OA treatment group (Figure 4(a)). The upstream regulation sequences among these 87 genes shared common predicted motif for Nkx-2.5 binding. The frequency matrix of this motif was shown. TTAATTG was the most frequent pattern (Figure 4(b)). The upstream regulatory region of a gene may feature multiple binding sites for a given transcription factor; we identified the genes with the highest number of possible Nkx-2.5 binding sites. They included FoxP1, Elav14, Zfp536, Erc2, and Runx1, among others (Figure 4(c)). We noted that many of these target genes with multiple binding site for Nkx-2.5 were reported to be involved in NSC function. We confirmed mRNA expression changes of FoxP1, Elav14, Zfp536, Erc2, and Runx1 by quantitative real-time PCR. The assay showed highly consistent results with DNA microarray measurement (Figure 4(d)).

### 3.5. Nkx-2.5 Mediated the Effects of OA on NSCs

**Figure 5:**
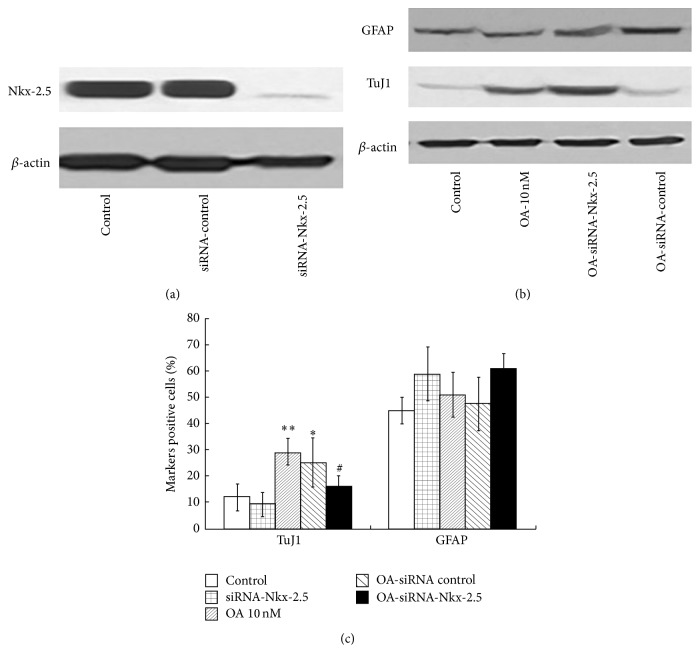
The siRNA of Nkx-2.5 was added to NSCs; 24 h later, cells were subjected to Western blotting analysis, indicating a marked knock-down of Nkx-2.5 expression (a). OA was added to the cells with silence of Nkx-2.5. The TuJ1 and GFAP protein expression was detected with Western blotting (b). Meanwhile, the immunofluorescence analysis of the percentage of TuJ1 and GFAP-positive cells was performed (*n* = 4). Results showed OA treatment resulted in significantly increase of the percentage of TuJ1-positive cells. However most effects of OA were abolished in OA-siRNA-Nkx-2.5 (*n* = 3; *P* < 0.05 versus OA 10 nM group), while the percentage did not significantly change in OA-siRNA-control (b and c). Scale bars: 100 *μ*m. Results were expressed as mean ± SD from 4 independent experiments. ^*^ *P* < 0.05  ^**^ *P* < 0.01 versus control; ^#^ *P* < 0.05 versus OA 10 nM.

In order to detect whether Nkx-2.5 is an essential mediator for the effects of OA, Nkx-2.5 was silenced with siRNA. After siRNA, Nkx-2.5 protein expression markedly decreased, while the control siRNA did not change the Nkx-2.5 protein expression (Figure 5(a)). siRNA of Nkx-2.5 did not significantly change the neuron-specific marker TuJ1 protein expression and the percentage of TuJ1-positive cells. This result suggested that Nkx-2.5 possibly has no constitutional effects on NSCs differentiation, consistent with a previous report in which Nkx-2.5 mainly acted as an essential transcription factor in myocardial cell lineage specification [34]. While 10 nM OA treatment resulted in significant increase in both TuJ1 protein expression and the percentage of TuJ1-positive cells (28.92 ± 5.0%), however most effects of OA were abolished by siRNA of Nkx-2.5 (*n* = 3; *P* < 0.05 versus OA 10 nM group) (Figures 5(b) and 5(c)). siRNA of Nkx-2.5 and/or OA treatment did not show significant influences on GFAP protein expression and GFAP-positive cells, possibly suggesting the specificity of action of OA on neural progenitors or from the high variance among experimental groups. Our results indicated that Nkx-2.5 is, at least in part, a mediator for the effects of OA in promoting differentiation of NSCs toward neurons.

## 4. Discussion

Single cells from the dissected neural tissue, when plated under appropriate conditions, form floating balls of cells termed neurospheres [35]. These are highly heterogeneous structures that contain true NSCs but also more restricted progenitors and even differentiated progeny. The presence of stem cells can be confirmed by dissociating these neurospheres into single cells and replating them. The more restricted progenitors and differentiated progeny have limited proliferation capacity. They either do not reform neurospheres or only reform very small neurospheres. By contrast, true NSCs have a strong capacity for self-renewal and reform neurospheres despite continual passaging. To definitively test the self-renewal potential of cells within the neurosphere cultures, clonal analysis is required. When single cells are plated at medium to high cell densities, cells or small neurospheres can adhere to each other and combine to form larger neurospheres [36, 37]. Typically these densities are used in drug screening assays, where it is not necessary that each neurosphere is derived from a single stem or progenitor cell. Many reports employ a low plating cell density of 5000 cells/mL or 1000 cells/mL to measure the self-renewal of neural stem cells [38, 39]. In our study, we plated the single cells at a density of 5000 cells/mL. About 5% plated of single cells reformed neurospheres, consistent with previous studies [39]. OA significantly inhibited the neurosphere formation and achieved the maximal effect at 10 nM. This decrease in neurosphere formation by OA may be derived from compromised proliferation of NSCs. To test this hypothesis, we measured proliferation after addition of OA using an EdU incorporation assay. OA significantly reduced EdU incorporation. The results above suggest that OA can inhibit the self-renewal of NSCs, possibly through inhibiting the proliferation of NSCs.

When NSCs differentiate, they gradually exit from cell cycle. Studies show that the length of the neural progenitor cell cycle is directly coupled to cell fate choices, since factors that shorten the cell cycle inhibit differentiative divisions, whereas those that lengthen the cell cycle promote differentiative divisions [40]. Based on the observation that the proliferative ability of NSCs was inhibited by OA, the effects of OA on the differentiation of NSCs were investigated. Immunocytochemistry analysis revealed that OA increased the percentage of TuJ1-positive cells and decreased the percentage of GFAP-positive cells. Western blotting showed OA increased TuJ1 protein expression and decreased GFAP expression. Mash1 is an essential transcription factor for promoting neurogenesis [41, 42]. To further confirm our findings, we determined the effects of OA on Mash1 protein expression and showed they were also elevated by OA. We also observed that, even in growth medium containing hrFGF and hrEGF, OA addition resulted in adherence of some neurospheres which showed neuron-like growth (data not shown). These results suggested that OA can effectively induce differentiation of NSCs into neurons.

Microarray analysis was used to screen for differentially expressed genes induced by OA. There were 80 genes upregulated by OA by more than 1.5-fold with *P* < 0.05, whilst 103 genes were downregulated. Transcription factors play an essential role in NSC maintenance and differentiation. We speculated that many of the differentially expressed genes may be targets of master regulatory transcription factors. We identified potential transcriptional factor binding sites in the upstream sequences of differentially expressed genes. Among the 183 genes differentially expressed by OA, 87 were predicted to have transcription factor binding sites for the Nkx-2.5 protein. Clustering analysis of these 87 genes showed expression changes could be classified into two groups that corresponded well to control and OA treatment. Nkx-2.5 is essential for myocardial cell lineage specification and development of the cardiac conduction system [34, 43, 44]. Nkx-2.5 also may play an important role in other organ or cell systems. Recently, overexpression of Nkx-2.5 in myoblasts was shown to result in expression of neuronal markers suggesting a role for this gene in neurogenesis [45]. Among the 87 genes predicted to be under the control of Nkx-2.5, there were several that contained multiple potential Nkx-2.5 binding sites including FoxP1, Elav14, Zfp536, Erc2, and Runx1. FoxP1 establishes columnar identity and connectivity of spinal motor neurons during mouse development [46, 47] and promotes the differentiation and/or maintenance of midbrain dopamine neurons [48]. Elavl4 is a gene coding for HuD, a member of mammalian ELAV/Hu proteins, and an RNA-binding protein. HuD expression is restricted to neurons. Within the nervous system, Hu proteins are one of the first markers of differentiated neurons [49]. ELAV/Hu proteins are also important in synaptic plasticity [50]. Zfp536, a recently identified zinc finger protein, is the most abundant in the brain, is expressed in the developing central nervous system and dorsal root ganglia, and is localized in the cerebral cortex, hippocampus, and hypothalamic area. Functional analyses provided evidence that Zfp536 is a negative regulator for neuron differentiation [51]. In this study, Zfp536 mRNA expression was significantly downregulated by OA, further supporting the effects of OA on neuronal differentiation. Runx1 may be another important target of Nkx-2.5. Runx1 plays an essential role in the differentiation of various cell types [52–54] and is involved in nerve cell innervation [55]. In summary, based on bioinformatics analysis and a review of the literature, we hypothesized that Nkx-2.5 may mediate the effects of OA.

In order to confirm our hypothesis about the role of Nkx-2.5, we used siRNA technique to silence the Nkx-2.5 expression in NSCs. Western blotting showed successful knocking down of most of the Nkx-2.5 expression. Silence of Nkx-2.5 did not increase or decrease the neuron-specific marker TuJ1 protein expression and the percentage of TuJ1-positive cells. This suggested that Nkx-2.5 possibly has no constitutional effects on NSCs differentiation. But we saw that, upon OA treatment, silence of Nkx-2.5 expression significantly, not fully, abolished the effects of OA on NSCs differentiation. Based on our results, we can not exclude other factors that played a role in effects of OA. Meanwhile, the relationship, even network relationship among Nkx-2.5 and other transcriptional factors such as FoxP1, Elav14, Zfp536, Erc2, and Runx1, will be our research focus in the near future. Even so, our results here indicated that Nkx-2.5 is, at least in part, a mediator for the effects of OA in promoting differentiation of NSCs toward neurons.

Neural stem cells (NSCs) can provide essential sources of engraftable neural cells for devastating diseases such as Alzheimer's disease [56], Parkinson's disease [57], and spinal cord injury [58]. One of the major challenges in the differentiation of NSCs is to increase the proportion of NSCs differentiating into neurons as opposed to glial cells. OA, derived from traditional Chinese herbs with long history of clinical application, may be a potential drug in improving neuronal differentiation and used in related diseases.

## 5. Conclusion

These results indicated that OA is an effective inducer for NSCs differentiation into neurons at least partially by Nkx-2.5-dependent mechanism.

## Supplementary Material

Table 1: Sequences of primers used in real-time PCR.
Table 2: KEGG pathways enrichment for the differentially regulated genes by OA.
Table 3: Gene ontology enrichment for the differentially regulated genes by OA.
Table 4: 87 Genes predicted under the control of Nkx-2.5 transcriptional factor among the total differentially regulated genes.
