# Supplementary material for: Oleanolic Acid Induces Differentiation of Neural Stem Cells to Neurons: An Involvement of Transcription Factor Nkx-2.5
Source: Stem Cells Int. 2015 Jul 9;2015:672312. doi: 10.1155/2015/672312 (PMC4512619; doi:10.1155/2015/672312)
Supplement: Supplementary file 1 — Table 1: Sequences of primers used in real-time PCR. Table 2: KEGG pathways enrichment for the differentially regulated genes by OA. Table 3: Gene ontology enrichment for the differentially regulated genes by OA. Table 4: 87 Genes predicted under the control of Nkx-2.5 transcriptional factor among the total differentially regulated genes. [file 672312.f1.zip › ╨┬╜¿╬─╝■╝╨/Supplemental Table 1.doc]

**Table 1 Sequences of primers used in real-time PCR**

| **Gene name** | **Gene ID** | **Forward primer** | **Reverse primer** | **Product size (bp)** |
| --- | --- | --- | --- | --- |
| Foxp1 | 108655 | GCAACCAGCTCTTCAGGTTC | CTCTTTCGGCTGTTTTCCAG | 241 |
| Elavl4 | 15572 | GGCAGAAGAAGCCATCAAAG | GTGATTGGGGAGAACCTGAA | 195 |
| Runx1 | 12394 | GCCAAGGATTTGCTCTGAAG | TTGGATCTTTGGGGTACAGC | 239 |
| Zfp536 | 243937 | GCTCCTGCCTCAACAAGAAC | TTCGGTGTCTAGCTCCTCGT | 176 |
| Erc2 | 238988 | GGGTCTTCCATGAACAGCAT | TCAGCTCAATGGTGAAGTGC | 250 |
