# Supplementary material for: Oleanolic Acid Induces Differentiation of Neural Stem Cells to Neurons: An Involvement of Transcription Factor Nkx-2.5
Source: Stem Cells Int. 2015 Jul 9;2015:672312. doi: 10.1155/2015/672312 (PMC4512619; doi:10.1155/2015/672312)
Supplement: Supplementary file 1 — Table 1: Sequences of primers used in real-time PCR. Table 2: KEGG pathways enrichment for the differentially regulated genes by OA. Table 3: Gene ontology enrichment for the differentially regulated genes by OA. Table 4: 87 Genes predicted under the control of Nkx-2.5 transcriptional factor among the total differentially regulated genes. [file 672312.f1.zip › ╨┬╜¿╬─╝■╝╨/Supplemental Table 2.doc]

**Table 2 KEGG pathways enrichment for the differentially regulated genes by OA**

| PathwayID | Definition | Genes | Fisher-Pvalue | FDR | Enrichment_Score |
| --- | --- | --- | --- | --- | --- |
| mmu04660 | T cell receptor signaling pathway - Mus musculus (mouse) | IL5,MAPK9,NCK2 | 0.01540422 | 0.1892572 | 1.81236 |
| mmu04672 | Intestinal immune network for IgA production - Mus musculus (mouse) | IL15RA,IL5 | 0.01886228 | 0.1892572 | 1.724406 |
| mmu05330 | Allograft rejection - Mus musculus (mouse) | 0610037M15RIK,IL5 | 0.02841402 | 0.1892572 | 1.546467 |
| mmu05320 | Autoimmune thyroid disease - Mus musculus (mouse) | 0610037M15RIK,IL5 | 0.04501572 | 0.1892572 | 1.346636 |
