# Supplementary material for: Oleanolic Acid Induces Differentiation of Neural Stem Cells to Neurons: An Involvement of Transcription Factor Nkx-2.5
Source: Stem Cells Int. 2015 Jul 9;2015:672312. doi: 10.1155/2015/672312 (PMC4512619; doi:10.1155/2015/672312)
Supplement: Supplementary file 1 — Table 1: Sequences of primers used in real-time PCR. Table 2: KEGG pathways enrichment for the differentially regulated genes by OA. Table 3: Gene ontology enrichment for the differentially regulated genes by OA. Table 4: 87 Genes predicted under the control of Nkx-2.5 transcriptional factor among the total differentially regulated genes. [file 672312.f1.zip › ╨┬╜¿╬─╝■╝╨/Supplemental Table 3.doc]

**Table 3 Gene ontology enrichment for the differentially regulated genes by OA**

| GO.ID | Term | Fold.Enrichment | Pvalue |
| --- | --- | --- | --- |
| GO:0006470 | protein amino acid dephosphorylation | 7.292926829 | 0.0023 |
| GO:0006687 | glycosphingolipid metabolic process | 21.44978479 | 0.0039 |
| GO:0045766 | positive regulation of angiogenesis | 20.25813008 | 0.0043 |
| GO:0016311 | dephosphorylation | 5.788037166 | 0.0052 |
| GO:0006664 | glycolipid metabolic process | 18.23231707 | 0.0053 |
| GO:0008152 | metabolic process | 1.375831641 | 0.0056 |
| GO:0032504 | multicellular organism reproduction | 3.29783513 | 0.0057 |
| GO:0048609 | reproductive process in a multicellular organism | 3.29783513 | 0.0057 |
| GO:0030031 | cell projection assembly | 7.292926829 | 0.0082 |
| GO:0007276 | gamete generation | 3.429275939 | 0.0085 |
| GO:0043412 | macromolecule modification | 2.016211999 | 0.0101 |
| GO:0022403 | cell cycle phase | 3.170837752 | 0.0122 |
| GO:0007283 | spermatogenesis | 3.661107846 | 0.0123 |
| GO:0048232 | male gamete generation | 3.661107846 | 0.0123 |
| GO:0008286 | insulin receptor signaling pathway | 11.39519817 | 0.0133 |
| GO:0019953 | sexual reproduction | 2.956591958 | 0.0167 |
| GO:0006464 | protein modification process | 1.947577009 | 0.0169 |
| GO:0000278 | mitotic cell cycle | 3.314966741 | 0.0181 |
| GO:0015758 | glucose transport | 9.595956354 | 0.0185 |
| GO:0008645 | hexose transport | 9.349906191 | 0.0194 |
| GO:0015749 | monosaccharide transport | 9.116158537 | 0.0203 |
| GO:0006639 | acylglycerol metabolic process | 8.893813206 | 0.0213 |
| GO:0006638 | neutral lipid metabolic process | 8.480147476 | 0.0233 |
| GO:0006662 | glycerol ether metabolic process | 8.480147476 | 0.0233 |
| GO:0022402 | cell cycle process | 2.728027492 | 0.0236 |
| GO:0006350 | transcription | 1.63171938 | 0.024 |
| GO:0032869 | cellular response to insulin stimulus | 8.287416851 | 0.0243 |
| GO:0046486 | glycerolipid metabolic process | 4.797978177 | 0.025 |
| GO:0030097 | hemopoiesis | 3.038719512 | 0.0252 |
| GO:0018904 | organic ether metabolic process | 7.92709438 | 0.0264 |
| GO:0045765 | regulation of angiogenesis | 7.92709438 | 0.0264 |
| GO:0000279 | M phase | 2.988904438 | 0.0268 |
| GO:0043687 | post-translational protein modification | 1.94336713 | 0.0273 |
| GO:0000280 | nuclear division | 3.506214822 | 0.028 |
| GO:0007067 | mitosis | 3.506214822 | 0.028 |
| GO:0000087 | M phase of mitotic cell cycle | 3.4728223 | 0.0288 |
| GO:0048285 | organelle fission | 3.440059825 | 0.0297 |
| GO:0071375 | cellular response to peptide hormone stimulus | 7.441762071 | 0.0297 |
| GO:0044238 | primary metabolic process | 1.303549838 | 0.0304 |
| GO:0045449 | regulation of transcription | 1.63131258 | 0.0321 |
| GO:0006629 | lipid metabolic process | 2.173748682 | 0.0322 |
| GO:0007049 | cell cycle | 2.167288805 | 0.0327 |
| GO:0048534 | hemopoietic or lymphoid organ development | 2.787816066 | 0.0346 |
| GO:0045941 | positive regulation of transcription | 2.469388317 | 0.0359 |
| GO:0006793 | phosphorus metabolic process | 1.915159356 | 0.0376 |
| GO:0006796 | phosphate metabolic process | 1.915159356 | 0.0376 |
| GO:0044255 | cellular lipid metabolic process | 2.436389809 | 0.0379 |
| GO:0022414 | reproductive process | 2.242991556 | 0.038 |
| GO:0000003 | reproduction | 2.231227614 | 0.0389 |
| GO:0048514 | blood vessel morphogenesis | 3.130011515 | 0.0398 |
| GO:0010628 | positive regulation of gene expression | 2.393739659 | 0.0407 |
| GO:0006355 | regulation of transcription, DNA-dependent | 1.819922757 | 0.0409 |
| GO:0006665 | sphingolipid metabolic process | 6.180446465 | 0.0417 |
| GO:0032868 | response to insulin stimulus | 6.180446465 | 0.0417 |
| GO:0045935 | positive regulation of nucleobase, nucleoside, nucleotide and nucleic acid metabolic process | 2.362719275 | 0.0429 |
| GO:0002520 | immune system development | 2.619585786 | 0.0433 |
| GO:0016481 | negative regulation of transcription | 2.619585786 | 0.0433 |
| GO:0006643 | membrane lipid metabolic process | 5.977808876 | 0.0443 |
| GO:0032870 | cellular response to hormone stimulus | 5.977808876 | 0.0443 |
| GO:0051252 | regulation of RNA metabolic process | 1.789076608 | 0.0453 |
| GO:0071495 | cellular response to endogenous stimulus | 5.881392604 | 0.0456 |
| GO:0051173 | positive regulation of nitrogen compound metabolic process | 2.303029525 | 0.0475 |
| GO:0007156 | homophilic cell adhesion | 5.697599085 | 0.0483 |
| GO:0019219 | regulation of nucleobase, nucleoside, nucleotide and nucleic acid metabolic process | 1.547425813 | 0.0487 |
