# Supplementary material for: Oleanolic Acid Induces Differentiation of Neural Stem Cells to Neurons: An Involvement of Transcription Factor Nkx-2.5
Source: Stem Cells Int. 2015 Jul 9;2015:672312. doi: 10.1155/2015/672312 (PMC4512619; doi:10.1155/2015/672312)
Supplement: Supplementary file 1 — Table 1: Sequences of primers used in real-time PCR. Table 2: KEGG pathways enrichment for the differentially regulated genes by OA. Table 3: Gene ontology enrichment for the differentially regulated genes by OA. Table 4: 87 Genes predicted under the control of Nkx-2.5 transcriptional factor among the total differentially regulated genes. [file 672312.f1.zip › ╨┬╜¿╬─╝■╝╨/Supplemental Table 4.doc]

**Table 1 87 Genes predicted under the control of Nkx-2.5 transcriptional factor among the total differentially regulated genes**

| SEQ_ID | GENE_NAME | GENE_ID | REGULATION | FOLD | P VALUE |
| --- | --- | --- | --- | --- | --- |
| AK086849 | B4galnt1 | 14421 | down | 2.891075 | 0.005961082 |
| BC032168 | Rap2b | 74012 | down | 2.545913 | 0.022933593 |
| AK013540 | Rcbtb2 | 105670 | down | 2.476134 | 0.003092449 |
| AK155262 | Runx1 | 12394 | up | 2.311227 | 0.007031526 |
| BC096589 | Zfp526 | 210172 | down | 2.207671 | 0.047374535 |
| BC055292 | Cds1 | 74596 | down | 2.064562 | 0.031910230 |
| AK090057 | Mapk9 | 26420 | down | 2.049880 | 0.007905034 |
| XM_974374 | Fam100a | 207740 | down | 1.966834 | 0.015980005 |
| AK030538 | Vti1a | 53611 | down | 1.945103 | 0.045481257 |
| AK052286 | Hisppd1 | 227399 | down | 1.944350 | 0.000143000 |
| BC019189 | Csn2 | 12991 | up | 1.922228 | 0.035476986 |
| BC105677 | Dnmt3b | 13436 | down | 1.895779 | 0.043447070 |
| AK049236 | Spg11 | 214585 | down | 1.882265 | 0.030474333 |
| AK052264 | Trak1 | 67095 | down | 1.880848 | 0.023114515 |
| AK172363 | Suclg2 | 20917 | up | 1.873624 | 0.048923820 |
| BC107383 | Rab9b | 319642 | down | 1.870928 | 0.049499087 |
| BC132543 | Fzd8 | 14370 | down | 1.865969 | 0.035639275 |
| BC113128 | D330045A20Rik | 102871 | down | 1.865264 | 0.007558773 |
| AK144648 | Cradd | 12905 | down | 1.848725 | 0.045642268 |
| BC012958 | Klf5 | 12224 | down | 1.846869 | 0.039469264 |
| DQ460221 | Elavl4 | 15572 | up | 1.839370 | 0.012713294 |
| BC019491 | Angptl3 | 30924 | up | 1.830931 | 0.043507013 |
| AK015067 | 4930403N07Rik | 73936 | up | 1.830155 | 0.042458850 |
| AK048211 | Adamts18 | 208936 | down | 1.823003 | 0.048819580 |
| BC039226 | Slc4a10 | 94229 | down | 1.812822 | 0.011532537 |
| AK032310 | Cspp1 | 211660 | down | 1.809367 | 0.037947852 |
| BC089360 | Pgbd1 | 319207 | down | 1.807607 | 0.014257123 |
| AB166747 | Trpm2 | 28240 | up | 1.805354 | 0.009716889 |
| BC145761 | Pag1 | 94212 | down | 1.805237 | 0.024934428 |
| AK173221 | Ppp4r4 | 74521 | down | 1.796921 | 0.002710675 |
| BC058593 | Senp7 | 66315 | down | 1.792150 | 0.029670004 |
| AK139083 | Klhl32 | 212390 | down | 1.788672 | 0.035474885 |
| X95761 | Arhgef2 | 16800 | down | 1.770209 | 0.023763705 |
| AK220392 | 6430573F11Rik | 319582 | up | 1.761333 | 0.036370806 |
| BC005746 | Klc4 | 74764 | down | 1.761069 | 0.018981066 |
| AK085965 | Prdxdd1 | 67939 | down | 1.738487 | 0.005554819 |
| AK049064 | Bcl7c | 12055 | down | 1.700711 | 0.045110647 |
| AK164546 | Cdh16 | 12556 | up | 1.689691 | 0.035305753 |
| AK012293 | Stxbp4 | 20913 | down | 1.688775 | 0.018696430 |
| AK047602 | Hecw2 | 329152 | up | 1.687607 | 0.012899855 |
| BC019407 | Ocel1 | 77090 | up | 1.683803 | 0.007459338 |
| AK030684 | BC023814 | 229503 | up | 1.683787 | 0.048758440 |
| AK015126 | Pitpnm2 | 19679 | down | 1.680288 | 0.046164628 |
| AK131832 | Rbpjl | 19668 | up | 1.671258 | 0.044908440 |
| AK045898 | Sdc3 | 20970 | up | 1.669873 | 0.030017132 |
| AK053370 | Mical2 | 320878 | up | 1.662320 | 0.049405962 |
| AK169248 | Ctdspl2 | 329506 | down | 1.661358 | 0.026628980 |
| AK034256 | Xdh | 22436 | up | 1.660176 | 0.042976190 |
| AF129509 | Ptprr | 19279 | up | 1.654522 | 0.045686215 |
| BC079566 | Grm1 | 14816 | down | 1.652125 | 0.031335168 |
| BC025573 | 2310014H01Rik | 76448 | down | 1.651358 | 0.032044183 |
| BC046790 | Arsk | 77041 | up | 1.649334 | 0.036347980 |
| AK049843 | Spock3 | 72902 | down | 1.649183 | 0.043916956 |
| AK028100 | Adk | 11534 | down | 1.644791 | 0.012397110 |
| BC079564 | Cnr1 | 12801 | down | 1.636479 | 0.010063274 |
| AK014480 | Dbf4 | 27214 | down | 1.634843 | 0.040585034 |
| AK045522 | Zbtb7c | 207259 | up | 1.634011 | 0.044568975 |
| BC006617 | Blvrb | 233016 | down | 1.633960 | 0.048293363 |
| AK170142 | Ssh2 | 237860 | down | 1.632708 | 0.014647387 |
| BC046961 | Zscan2 | 22691 | down | 1.631080 | 0.041149862 |
| BC021425 | Stc1 | 20855 | down | 1.629814 | 0.039597724 |
| AK011980 | Ggps1 | 14593 | down | 1.629610 | 0.026306542 |
| AK087631 | Irf6 | 54139 | up | 1.627388 | 0.014351843 |
| AK044218 | Kcnj6 | 16522 | up | 1.626098 | 0.009291704 |
| AK033491 | Kctd3 | 226823 | down | 1.611936 | 0.007291947 |
| BC115428 | Klhl30 | 70788 | up | 1.611465 | 0.040938120 |
| AK183781 | Zdhhc16 | 74168 | down | 1.594361 | 0.006300065 |
| AK039787 | Gab3 | 210710 | up | 1.589479 | 0.014780683 |
| BC125366 | Il5 | 16191 | up | 1.585042 | 0.048765995 |
| AK164743 | Nup98 | 269966 | down | 1.571062 | 0.035860950 |
| AK142740 | Cyp2u1 | 71519 | down | 1.565267 | 0.047552690 |
| AK014743 | Iah1 | 67732 | up | 1.563946 | 0.016314404 |
| AK044901 | Cnnm4 | 94220 | up | 1.559541 | 0.039750665 |
| AK043389 | Mettl4 | 76781 | down | 1.557717 | 0.026709747 |
| AK015641 | Crem | 12916 | up | 1.555837 | 0.039147925 |
| AK012646 | Grb10 | 14783 | down | 1.549436 | 0.048212986 |
| AK042083 | Msh5 | 17687 | down | 1.547711 | 0.049344048 |
| BC061175 | Tssk2 | 22115 | up | 1.541146 | 0.030523732 |
| AF339104 | Foxp1 | 108655 | down | 1.538105 | 0.026815130 |
| AK041609 | Epm2a | 13853 | down | 1.525503 | 0.035137504 |
| BC036162 | Irf2bp1 | 272359 | down | 1.519363 | 0.040709410 |
| BC033594 | Zfp536 | 243937 | down | 1.510483 | 0.027404413 |
| BC117072 | 4933400C05Rik | 328019 | up | 1.504214 | 0.025023881 |
| BC092141 | Ndufc1 | 66377 | down | 1.503900 | 0.030939799 |
| AY356531 | Erc2 | 238988 | down | 1.503623 | 0.034229580 |
| AK089749 | D8Ertd82e | 244418 | up | 1.501483 | 0.031189183 |
| AK007017 | Ccdc91 | 67015 | up | 1.500944 | 0.010063086 |
